# Quantifying and Interpreting the Association between Early-Life Gut Microbiota Composition and Childhood Obesity

**DOI:** 10.1128/mBio.02787-18

**Published:** 2019-02-12

**Authors:** Shirin Moossavi, Meghan B. Azad

**Affiliations:** a Medical Microbiology and Infectious Diseases, University of Manitoba, Winnipeg, Manitoba, Canada; b Children’s Hospital Research Institute of Manitoba, Pediatrics and Child Health, Winnipeg, Manitoba, Canada; c Developmental Origins of Chronic Diseases in Children Network (DEVOTION), Winnipeg, Manitoba, Canada; d Digestive Oncology Research Center, Digestive Disease Research Institute, Tehran University of Medical Sciences, Tehran, Iran; Yale School of Public Health

**Keywords:** infant, microbiota, obesity

## LETTER

We read with interest the recent article by Stanislawski et al. (1) on the association of early-life gut microbiota composition with child body mass index (BMI) in the exquisitely phenotyped Norwegian Microbiota Study (NoMIC) birth cohort. The authors observed that gut microbiota compositions among 165 Norwegian children at 10 days and 2 years of age were significantly associated with BMI at 12 years. They reported *R*^2^ values from random-forest analyses and interpreted these values as the proportion of BMI variation explained by the early-life gut microbiota, concluding that over 50% of variation in BMI at 12 years was explained by gut microbiota at 2 years (1).

Although we certainly agree that early-life events have long-term health consequences and that gut microbiota contribute to energy harvest and weight gain, there are many other contributing factors to weight gain trajectories throughout childhood, and it seems biologically implausible that 50% of BMI variation in mid-childhood could be explained by gut microbiota during infancy. By comparison, in conventional regression analyses, we have found that a far smaller proportion of variation in BMI is explained by established obesity risk factors. For example, among over 2,600 infants in the Canadian Healthy Infant Longitudinal Development (CHILD) cohort, we observed that only 10.3% of variation in BMI at 1 year was collectively explained by maternal BMI, race, socioeconomic status, smoking, delivery mode, parity, breastfeeding, infant sex, birth weight, and gestational age (*R*^2^ = 0.103 in the fully adjusted model reported in reference 2).

We appreciate that the authors statistically controlled for several factors that could disrupt the infant gut microbiota (i.e., delivery mode, breastfeeding, and antibiotics). However, other key risk factors of childhood obesity, including maternal BMI, smoking, child diet, and physical activity, were not accounted for (3). Although some of these factors come into play long after the “exposure” to early-life microbiota, they are relevant to include as a reference point for the observed microbiota effect sizes. It would also be useful to account for infant sex because important sex differences have been reported in the association of infant weight gain trajectories with maternal BMI (4) and breastfeeding (5).

Finally, we applaud the authors for defining conceptual frameworks and causal pathways, where they conceive breastfeeding, mode of delivery, antibiotics, and gestational age as confounders and/or mediators of the association between gut microbiota and BMI, in accordance with previous observations from the CHILD cohort (6). We also envision another scenario where breastfeeding could play a moderating (rather than a mediating) role within the defined theoretical framework, potentially mitigating the obesogenic effect of maternal obesity and other detrimental early-life exposures. It would be very interesting to test this hypothesis in the NoMIC cohort, which is among the longest-running birth cohorts with early-life microbiota and longitudinal health trajectory data. We hope that these suggestions will stimulate new research hypotheses and fruitful scientific dialogue on the impact of gut microbiota during infancy on weight gain trajectories later in life.
